# Impaired performance of SARS-CoV-2 antigen-detecting rapid diagnostic tests at elevated and low temperatures

**DOI:** 10.1016/j.jcv.2021.104796

**Published:** 2021-05

**Authors:** Verena Haage, Edmilson Ferreira de Oliveira-Filho, Andres Moreira-Soto, Arne Kühne, Carlo Fischer, Jilian A. Sacks, Victor Max Corman, Marcel A. Müller, Christian Drosten, Jan Felix Drexler

**Affiliations:** a Institute of Virology, Charité-Universitätsmedizin Berlin, Corporate Member of Freie Universität Berlin, Humboldt-Universität zu Berlin, Berlin, Germany; b German Centre for Infection Research (DZIF), Associated Partner Charité-Universitätsmedizin Berlin, Berlin, Germany; c Foundation for Innovative New Diagnostics (FIND), Geneva, Switzerland

**Keywords:** SARS-CoV-2, Rapid antigen test, Temperature stability, Sensitivity, Specificity, Tropics, Winter

## Abstract

Antigen-detecting rapid diagnostic tests (Ag-RDTs) can complement molecular diagnostics for COVID-19. The recommended temperature for storage of SARS-CoV-2 Ag-RDTs ranges between 2−30 °C. In the global South, mean temperatures can exceed 30 °C. In the global North, Ag-RDTs are often used in external testing facilities at low ambient temperatures.

We assessed analytical sensitivity and specificity of eleven commercially-available SARS-CoV-2 Ag-RDTs using different storage and operational temperatures, including short- or long-term storage and operation at recommended temperatures or at either 2−4 °C or at 37 °C. The limits of detection of SARS-CoV-2 Ag-RDTs under recommended conditions ranged from 1.0×10^6^- 5.5×10^7^ genome copies/mL of infectious SARS-CoV-2 cell culture supernatant. Despite long-term storage at recommended conditions, 10 min pre-incubation of Ag-RDTs and testing at 37 °C resulted in about ten-fold reduced sensitivity for five out of 11 SARS-CoV-2 Ag-RDTs, including both Ag-RDTs currently listed for emergency use by the World Health Organization. After 3 weeks of storage at 37 °C, eight of the 11 SARS-CoV-2 Ag-RDTs exhibited about ten-fold reduced sensitivity. Specificity of SARS-CoV-2 Ag-RDTs using cell culture supernatant from common respiratory viruses was not affected by storage and testing at 37 °C, whereas false-positive results occurred at outside temperatures of 2−4 °C for two out of six tested Ag-RDTs, again including an Ag-RDT recommended by the WHO.

In summary, elevated temperatures impair sensitivity, whereas low temperatures impair specificity of SARS-CoV-2 Ag-RDTs. Consequences may include false-negative test results at clinically relevant virus concentrations compatible with transmission and false-positive results entailing unwarranted quarantine assignments. Storage and operation of SARS-CoV-2 Ag-RDTs at recommended conditions is essential for successful usage during the pandemic.

## Highlights

• Analytical sensitivity of SARS-CoV-2 Ag-RDTs ranges from 1.0 × 10^6^-5.5 × 10^7^ copies/mL.
• Even short-term exposure to 37 °C reduces sensitivity of SARS-CoV-2 Ag-RDTs.
• Elevated temperatures impair sensitivity at clinically relevant virus concentrations.
• Low temperatures limit SARS-CoV-2 Ag-RDT specificity.
• Storage and operation of SARS-CoV-2 Ag-RDTs at recommended conditions is essential.

## 1 Introduction

**Fig. 1:**
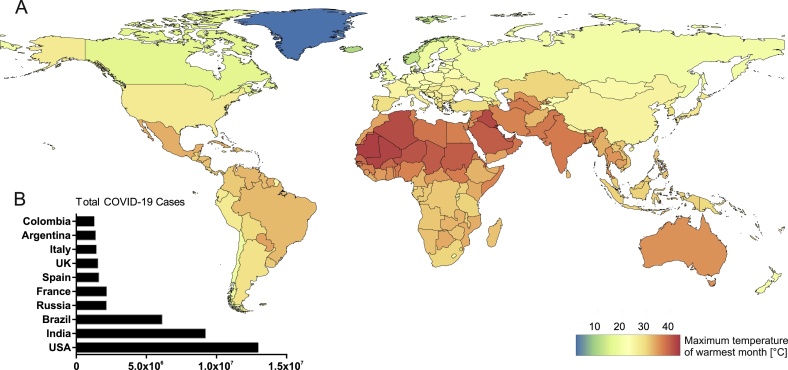
**COVID-19 case numbers and maximum temperatures globally. A**. World map representing global temperature distribution based on maximum temperature of the warmest month (°C) freely available from WorldClim 2 [8]. **B.** Graph represents total COVID-19 cases in the ten most affected countries globally by 25^th^ of November 2020 [9].

Advantages of SARS-CoV-2 antigen-detecting rapid diagnostic tests (Ag-RDTs) include fast results and their applicability on site without dependence on laboratory settings. With a constantly growing number of commercially available Ag-RDTs on the global market, the number of studies validating Ag-RDTs from different manufacturers is increasing rapidly [[1], [2], [3], [4], [5], [6], [7]]. However, none have interrogated the performance of Ag-RDTs under conditions that differ from supplier-recommended storage and operation conditions (2−30 °C), such as those observed in tropical settings where ambient temperatures routinely exceed 30 °C (Fig. 1A). This is challenging because tropical regions are strongly affected by the SARS-CoV-2 pandemic as evident from total cases reported from India, Brazil, Argentina, and Colombia, four out of the ten most affected countries worldwide by November 2020 (Fig. 1B).

On the other hand, the global North was heavily affected by the second wave of the COVID-19 pandemic during November 2020-February 2021 [10,11]. To manage testing demand, different actors have opened external testing stations such as diagnostic streets or drive-through facilities in urban settings [12]. These facilities are often of provisional nature, for example in the form of unheated tents. In the winter months, temperatures in Europe or the U.S. can range from -10 °C to 10 °C [13,14], well below the recommended operating temperatures of most Ag-RDTs. Most manufacturers of SARS-CoV-2 Ag-RDTs specify storage conditions between 2−30 °C, but stipulate that tests be equilibrated to room temperature (15−30 °C) at the time of use to guarantee performance. With temperatures around freezing point during the winter months, unheated testing facilities cannot always comply with these conditions. Temperature tolerance of SARS-CoV-2 diagnostic tools or environmental stability requirements have been previously discussed as hurdles to be addressed according to the World Health Organization (WHO) [15,16].

To validate the performance of SARS-CoV-2 Ag-RDTs in both, tropical and cold settings, we compared analytical sensitivity and specificity using recommended conditions and either elevated or low temperatures.

## 2 Materials and methods

### 2.1 Analytical sensitivity

**Fig. 2:**
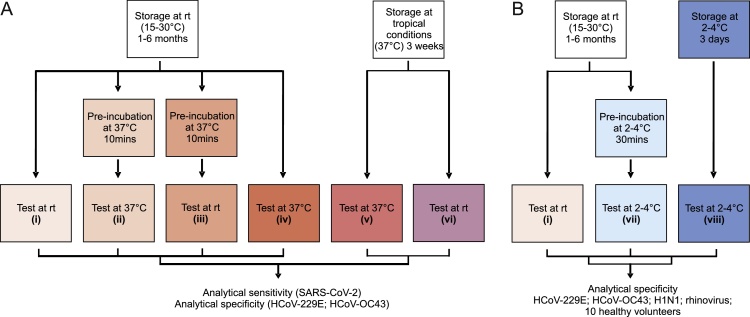
**Experimental setup. A. Validation of SARS-CoV-2 Ag-RDTs at elevated temperatures.** (i): storage at recommended conditions (room temperature (rt); 15-30 °C) for 1-6 months and test operation at recommended conditions (rt; 15-30 °C). (ii): storage at recommended conditions (rt; 15-30 °C) for 1-6 months, 10 min pre-incubation of tests at 37 °C prior to operation at 37 °C so as to mimic recommended storage of kits prior to test usage under non air-conditioned conditions in tropical settings. (iii): storage at recommended conditions (rt; 15-30 °C) for 1-6 months, 10 min pre-incubation of tests at 37 °C prior to operation at room temperature. (iv): storage at recommended conditions (rt; 15-30 °C) for 1-6 months and test operation at 37 °C. Settings (v) and (vi) covered storage under tropical conditions (37 °C) for 3 weeks followed by either test operation at 37 °C to mimic non air-conditioned storage and test operation in tropical settings (v) or test operation at room temperature to mimic non air-conditioned storage and test application at room temperature (vi). **B. Validation of SARS-CoV-2 Ag-RDTs at low temperatures.** (i): storage at recommended conditions (room temperature (rt); 15-30 °C) for 1-6 months and test operation at recommended conditions (rt; 15-30 °C). (vii): storage of tests at recommended conditions, pre-incubation of tests for 30 min at cold temperatures (2-4 °C) and operation at cold temperatures (2-4 °C). (viii): storage of tests at 2-4 °C for 3 days followed by testing at 2-4 °C. rt = room temperature.

SARS-CoV-2 (BetaCoV/Munich/ChVir984/2020) was grown on Vero E6 cells (C1008; African green monkey kidney cells), maintained in DMEM (10 % FCS) at 37 °C with 5% CO2. For quantification, viral RNA was extracted using the QIAamp Viral RNA Mini Kit (Qiagen, Hilden, Germany) and quantified using photometrically quantified *in vitro*-transcribed RNA standards [17,18]. For determining the limit of detection (LOD), SARS-CoV-2 stock (2.2×10^9^ copies/mL) was serially diluted in plain DMEM and 5 μL per dilution were added to the extraction buffer of the respective kit for validation experiments. For Coris COVID-19 Ag Respi-Strip, 5 μL of SARS-CoV-2 supernatant were added to 95 μL of PBS to reach the required sample volume of 100 μL prior to addition of LY-S buffer. Validation experiments were performed in triplicates for a subset of tests at recommended conditions (Fig. 2, setting (i)) initially, with all three replicates showing the same result (Supplementary Table S1). Consequently, due to a limited number of available tests, experiments were performed in duplicates. LOD was defined as the lowest dilution at which both replicates were positive. A dilution factor correction was applied based on the volume of extraction buffer (range: 100−500 μl) provided by each SARS-CoV-2 Ag-RDT kit.

### 2.2 Analytical specificity

Specificity for tropical conditions was assessed using cell culture supernatant of the ubiquitous human coronaviruses HCoV-229E (2.9×10^7^ copies/mL) and HCoV-OC43 (1.0×10^6^ copies/mL). 5 μL of viral cell culture supernatant were added to proprietary lysis buffer except for Coris COVID-19 Ag Respi-Strip as described above.

Specificity for cold conditions was tested using cell culture supernatant of common respiratory viruses including HCoV-229E, HCoV-OC43, influenza virus A H1N1 (7.8×10^6^ copies/mL) and rhinovirus A (2.2×10^6^ copies/mL). 20 μL of viral cell culture supernatant were added to proprietary lysis buffer or as an internal control 20 μL of lysis buffer were directly applied to test cassettes for validation experiments. Viral concentrations were selected according to the guidelines on analytical specificity testing for SARS-CoV-2 Ag-RDTs published by the German Federal institute for vaccines and biomedicines [19].

### 2.3 Healthy SARS-CoV-2 negative subjects

Additionally, ten healthy laboratory members who previously volunteered for a SARS-CoV-2 Ag-RDT validation study were tested [1]. Healthy volunteers were without symptoms of respiratory tract infection and tested negative for SARS-CoV-2 by RT-qPCR [20]. All subjects received instructions on self-sampling, recently shown to be a reliable alternative to nasopharyngeal swabs taken by professional healthcare workers for Ag-RDTs [21]. Swabs were dissolved immediately in 1 mL PBS and 20 μL of PBS containing respiratory material from study participants were added to proprietary buffer for testing.

### 2.4 Interpretation of test results

For tests with visual readout, results in the form of a band were scored by two researchers independently and in case of discrepancy a third person was consulted to reach a final decision (reader-based tests: Bioeasy 2019-nCoV Ag and ichroma - COVID-19 Ag). Results were defined as borderline when a weak, discontinuous band or smear was observed that could not be clearly defined as a positive or negative result.

### 2.5 World heat map

Data of maximum temperatures of the warmest month (°C) on country level at the spatial resolution of 2.5 min were obtained from WorldClim 2 [8]. R package 'exactextractr' version 4.0.2 was used to calculate national means. Data on COVID-19 cases were obtained from Worldmeter [9] and visualized using the GraphPad Prism software version 9.1.0.

## 3 Results

At present, there are at least 139 SARS-CoV-2 Ag-RDTs commercially available [22], from which 11 were selected for temperature stability validation at elevated temperatures based on the availability of clinical performance data [1] and manufacturing by leading suppliers implying availability on the global market (Table 1).

**Table 1:** Overview of SARS-CoV-2 antigen-detecting rapid diagnostic tests included in the study.

ID	Test	Manufacturer	Tested at	Lot No.
I	Panbio™ COVID-19 Ag Rapid Test	Abbott Laboratories	37 °C; 2−4 °C	41ADF012A
II	ActivXpress + COVID-19 Antigen Complete Testing Kit	Edinburgh Genetics	37 °C; 2−4 °C	AG20200905
III	Bioeasy 2019-nCoV Ag Fluorescence Rapid Test Kit	Shenzhen Bioeasy Biotechnology Co., Ltd	37 °C	2003N406
IV	Clinitest Rapid COVID-19 Antigen Test	Siemens Healthineers	37 °C	2010184
V	Covid.19 Ag Respi-Strip	Coris BioConcept	37 °C	43871J2008 43760I2015
VI	COVID-19 Ag	Genedia	37 °C; 2−4 °C	643X2005
VII	ichroma - COVID-19 Ag	Boditech Med	37 °C; 2−4 °C	SRQHA27
VIII	COVID-19 Antigen Rapid Test Kit	JOYSBIO (Tianjin) Biotechnology Co., Ltd.	37 °C; 2−4 °C	2020092409
IX	NowCheck COVID-19 Ag test	BIONOTE INC.	37 °C	1901D002 Code GEN
X	SARS-CoV-2 Rapid Antigen Test	Roche Diagnostics*	37 °C; 2−4 °C	QCO3020083 QCO390003I/Sub:I-2 QCO390011A/Sub:A-2
XI	STANDARD Q COVID-19 Test	SD Biosensor, Inc.	37 °C	QCO3020040A

* equals STANDARD Q COVID-19 Test by SD Biosensor, Inc.

Subsequently, analytical performance of the selected SARS-CoV-2 Ag-RDTs was assessed following storage and application of tests under recommended conditions as well as elevated temperatures (termed tropical conditions henceforth), using six different experimental settings (Fig. 2A). The tested conditions were defined by different combinations of storage time (short- and long-term storage) at either recommended (15-30 °C) or elevated temperatures (37 °C) and subsequent test operation at either recommended (15-30 °C) or elevated temperatures (37 °C).

First, we determined analytical sensitivity at recommended conditions by determining the limit of detection (LOD) of SARS-CoV-2 Ag-RDTs when stored and operated at room temperature (15-30 °C; setting (i); Fig. 2) . The dilution-factor corrected LODs for validated SARS-CoV-2 Ag-RDTs ranged from 1.0×10^6^ copies/mL to 5.5×10^7^ copies/mL of SARS-CoV-2 cell culture supernatant (Table 2). Even though we used a relatively low number of replicates, those LODs were consistent with previously published virus concentrations for validation of SARS-CoV-2 Ag-RDTs using clinical samples [1], suggesting robustness of our data. Our data also highlight profound differences in analytical sensitivity of up to 50-fold for SARS-CoV-2 Ag-RDTs from different manufacturers.

**Table 2:** Analytical sensitivity of SARS-CoV-2 antigen-detecting rapid diagnostic tests at recommended storage and tropical test conditions.

SARS-CoV-2 (Cps/mL)	I	II	III	IV	V	VI	VII	VIII	IX	X	XI
2.2 × 10^9^	++	++	++	++	++	++	++	++	++	++	++
5.9 × 10^8^	++	++	++	++	?	?	++	++	++	++	++
8.6 × 10^7^	++	++	–	+	–	–	++	+	++	+	++
7.2 × 10^6^	–	–	–	–	–	–	–	–	–	–	–
Corrected SARS-CoV-2 LOD (Cps/mL)	1.4×10^6^	1.7×10^6^	5.9×10^6^	1.3×10^6^	5.5×10^7^	3.2×10^7^	1.0×10^6^	1.7×10^6^	8.4×10^6^	1.2×10^6^	1.2×10^6^

I: Abbott; II ActivXpress; III Bioeasy; IV Clinitest; V Coris; VI Genedia; VII ichroma; VIII JOYSBIO; IX NowCheck; X Roche; XI Standard Q. ++ positive; + weak positive; - negative;? unclear result. LOD: limit of detection. Cps, Genome copies.

**Fig. 3:**
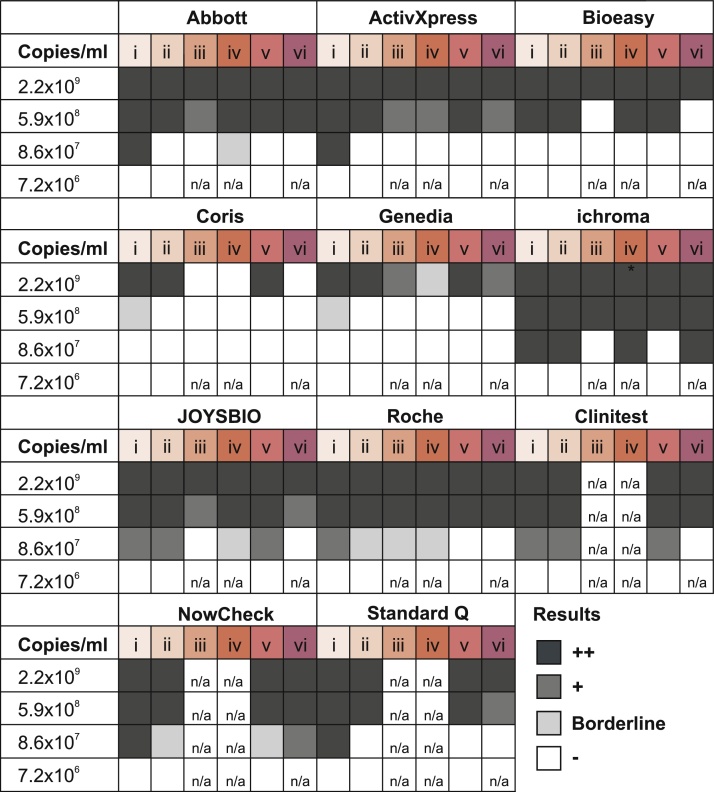
**Sensitivity of SARS-CoV-2 Ag-RDTs decreases at elevated temperatures.**Analytical sensitivity of SARS-CoV-2 rapid antigen tests upon different storage and operation conditions; i: storage and test operation at recommended conditions (rt; 15-30 °C); ii: storage at recommended conditions (rt; 15-30 °C), 10 min pre-incubation at 37 °C prior to operation at 37 °C; iii: storage at recommended conditions (rt; 15-30 °C), 10 min pre-incubation at 37 °C prior to operation at recommended conditions (rt; 15-30 °C); iv: storage at recommended conditions (rt; 15-30 °C) and test operation at 37 °C; v: storage and testing at 37 °C; vi: storage at 37 °C and testing at recommended conditions (rt; 15-30 °C); ++ positive; + weak positive; borderline: unclear result; - negative;. rt: room temperature. n/a: data not available.

We then assessed analytical sensitivity of SARS-CoV-2 Ag-RDTs after long-term storage at recommended conditions (15-30 °C; 1-6 months) followed by short-term exposure to 37°C (10 min) and test operation at either 37 °C (setting (ii); Fig. 2A) or at recommended temperatures (15-30 °C; setting (iii); Fig. 2A). The analytical sensitivity of about half of the evaluated SARS-CoV-2 Ag-RDTs (five out of eleven; 45 %) was already compromised by about ten-fold when tests were stored under recommended conditions but exposed to 37 °C for only ten minutes prior to testing at 37 °C (condition ii; Fig. 3; for LOD refer to Supplementary Table S2). This effect was even more pronounced when tests were stored under recommended conditions but exposed to 37 °C for ten minutes prior to testing at recommended temperatures (15-30 °C) (condition iii; Fig. 3), as all eight tested kits (three other kits were not available in sufficient numbers for testing this condition) showed an about 10-fold reduced sensitivity under this experimental setting. We also assessed test performance when Ag-RDTs were stored at recommended temperatures (15-30 °C) followed by direct operation at 37 °C (setting (iv); Fig. 2A). Even under these conditions we found an about ten-fold reduction in analytical sensitivity for six out of eight tested kits (75%; three other kits were not available in sufficient numbers for testing this condition), underlining the importance of test operation at recommended conditions (condition iv; Fig. 3). We additionally tested analytical sensitivity of SARS-CoV-2 Ag-RTDs after long-term storage at 37 °C (3 weeks) followed by test operation at either 37 °C (setting (v); Fig. 2A) or at recommended temperatures (15-30 °C; setting (vi); Fig. 2A). After 19–21 days of storage at 37 °C and testing at 37 °C (condition v, Fig. 3) or testing at recommended temperatures (15-30 °C) (condition vi, Fig. 3), eight out of the total eleven SARS-CoV-2 Ag-RDTs (73 %) showed an about ten-fold reduction in analytical sensitivity when compared to recommended temperatures. In sum, those data indicate that even short-term exposure of SARS-CoV-2 Ag-RDTs to elevated temperatures affects their sensitivity and that multiple temperature shifts might more seriously affect test sensitivity.

Additionally, analytical specificity of SARS-CoV-2 Ag-RDTs under recommended storage and operating conditions and under tropical storage and operating conditions at 37 °C (settings (i) and (v); Fig. 2A) was examined by testing for cross-reactivity with the ubiquitous human coronaviruses HCoV-229E and HCoV-OC43 [23,24]. SARS-CoV-2 Ag-RDTs showed no cross-reactivity with HCoV-229E or HCoV-OC43 upon storage and testing at elevated temperatures (Table 3).

**Table 3:** Analytical specificity of SARS-CoV-2 antigen-detecting rapid diagnostic tests at recommended and tropical storage and test operation conditions.

Condition	Virus	Cps/mL	I	II	III	IV	V	VI	VII	VIII	IX	X	XI
i	HCoV-229E	2.9×10^7^	–	–	–	–	–	–	–	–	–	–	–
v			–	–	–	–	–	–	–	–	–	–	–
(i)	HCoV-OC43	1.0×10^6^	–	–	–	–	–	–	–	–	–	–	–
v			–	–	–	–	–	–	–	–	–	–	–

I: Abbott; II ActivXpress; III Bioeasy; IV Clinitest; V Coris; VI Genedia; VII ichroma; VIII JOYSBIO; IX NowCheck; X Roche; XI Standard Q.+ positive; - negative. Cps, Genome copies. Tests were performed in duplicates.

**Fig. 4:**
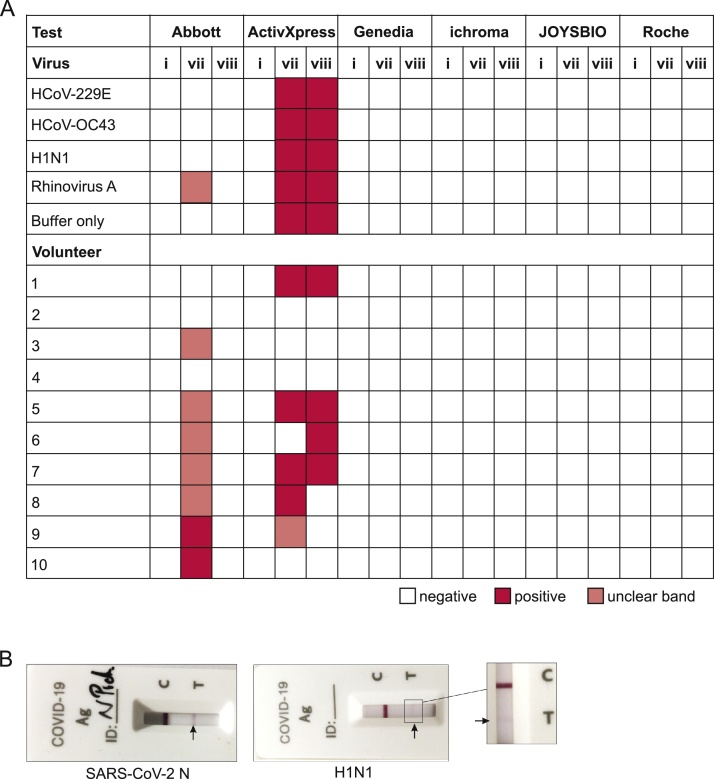
**Specificity of SARS-CoV-2 Ag-RDTs decreases at low temperatures. A.**i: storage and test operation at recommended conditions (rt; 15-30 °C); vii: storage at recommended conditions (rt; 15-30 °C), 30 min pre-incubation of tests at 2-4 °C prior to test operation at 2-4 °C; viii: storage at 2-4 °C for 3 days and testing at 2-4 °C; red: positive; white: negative; salmon: weak band, result unclear. rt: room temperature. Tests were performed in duplicates. **B**. Example for observed cross-reactivity of the ActivXpress test with Influenza virus A H1N1 and SARS-CoV-2 nucleoprotein as positive control (SARS-CoV-2-N; 5 μg/mL) when tested under condition vii: storage at recommended conditions (rt; 15-30 °C), 30 min pre-incubation of tests at 2-4 °C prior to test operation at 2-4 °C.

As the national COVID-19 reference laboratory in Germany, we have been contacted by multiple outside testing facilities across Germany reporting an unusually high number of positive SARS-CoV-2 Ag-RDTs. In order to validate the specificity of SARS-CoV-2 Ag-RDTs when operated at low outside temperatures, we compared Ag-RDT performance after recommended storage and short-term exposure (30 min) to 2−4 °C prior to testing at 2−4 °C (setting (vii); Fig. 2B) as well as after storage at 2−4 °C followed by test operation at 2−4 °C (setting (viii); Fig. 2B) to Ag-RDT specificity when stored and operated under recommended conditions (setting (i); Fig. 2B). We hereto selected a subset of six Ag-RDTs for reasons of scarcity of tests and urgency to conduct the testing under the weather conditions that prevailed at the time of physicians’ reports from external testing stations (Table 1). Two of the six SARS-CoV-2 Ag-RDTs showed impaired specificity (Fig. 4A) when stored at room temperature, but when exposed to 2−4 °C for 30 min prior to testing at 2−4 °C (condition vii; Fig. 4A) as cross-reactivity with common respiratory viruses, and false-positive results occurred in healthy volunteers in the form of weak, but clearly visible bands (Fig. 4B). In one test (test I), unspecific reactivity was only observed upon short-term exposure to 2−4 °C followed by test operation at 2−4 °C (condition vii; Fig. 4A), but not after long-term storage at 2−4 °C (condition viii; Fig. 4A). In contrast, the other test which showed non-specific results (test II) yielded almost identically unspecific results after both, short- and long-term storage at 2−4 °C and test operation at 2−4 °C (conditions vii and viii; Fig. 4A). On the one hand, those data highlight differences between test devices. On the other hand, our results may hint at effects of relatively rapid temperature changes on some tests for unknown reasons, potentially including environmental factors such as condensation. Results were reproducible and functionality of tests was confirmed by determining their LODs using serial dilutions of SARS-CoV-2 nucleoprotein (SARS-CoV-2-N) at recommended conditions as previously described [1].

## 4 Discussion

Our study highlights that temperature during storage and operation of SARS-CoV-2 Ag-RDTs affects test performance. First, even short-term exposure to elevated temperatures may compromise sensitivity of currently available SARS-CoV-2 Ag-RDTs. Our data are consistent with impaired sensitivity of other Ag-RDTs at elevated temperatures, including Malaria rapid diagnostic tests (MRDTs). An assessment of five MRDTs reported a 13%–53% decline in sensitivity for three of those MRDTs following 90 days of storage at 35 °C [25]. Moreover, an evaluation assessing temperature stability of dengue NS1 antigen-based RDTs at 35 °C showed a gradual decline in test sensitivity for seven out of eight tested dengue Ag-RTDs after storage for about 20 days at elevated temperatures [26]. Beyond storage, elevated temperatures during shipment can also affect Ag-RDT performance. Supply chains of MRDTs were studied in Burkina Faso, Senegal, Ethiopia, the Philippines and Cambodia, demonstrating regular exceeding of 30 °C during transport [27,28]. Consequently, the WHO recommends heat stability testing between 35 °C and 40 °C for MRDTs [29] and supply and delivery chains to tropical countries must contain adequate cold chains [30]. Our data thus imply a huge challenge to tropical countries with regard to adequate transportation and storage of SARS-CoV-2 Ag-RDTs. To guarantee temperature-regulated storage, a certain level of infrastructure is required, ideally including air-conditioned facilities with temperature monitors and secured power supply. Unfortunately, these requirements are not always realistic in resource-limited settings and appropriate concepts for adequate storage in remote areas without electricity and rudimentary infrastructure will be required.

Moreover, our study highlights that specificity of SARS-CoV-2 Ag-RDTs may be impaired when operating tests at temperatures below commonly recommended conditions, leading to false-positive results. These results were observed for certain test brands only, including one of the Ag-RDT currently listed for emergency use by the WHO [31,32], highlighting that each test may need to be considered specifically and broader validation of temperature robustness of SARS-CoV-2 Ag-RDTs should be performed. Of note, all tests studied here were shown to be highly specific when operated at recommended conditions in prior studies [1], underlining that impaired specificity is not a test-intrinsic problem but owed to test operation under conditions beyond those defined by the manufacturers. Our data imply that caution must be taken when offering SARS-CoV-2 Ag-RDT-based detection in settings lacking temperature control, including diagnostic streets, drive-through testing stations and self-use by untrained individuals at home[12]. Irrespective of the setting, compliance with the conditions recommended by the manufacturer are vital to ensure accurate testing [33]. As discussed by others, temperature stability guidelines for *in vitro* diagnostics exist, however there are currently no specific guidelines for the validation of Ag-RDTs regarding temperature stability [[34], [35], [36], [37]]. Common validation guidelines including environmental conditions could be a first step towards globally reliable diagnostics.

Our study is limited by focusing on analytical test performance for reasons of comparability of test results across the different conditions and based on limited access to clinical samples. An additional limitation of our study is the use of duplicates for some tests instead of a higher number of replicates, which was due to the limited availability of all tests included in the study. Further studies will be required to assess test performance upon storage and application in tropical as well as cold conditions using large numbers of clinical samples. Despite these limitations, our study presents a robust resource for further validation studies as a high number of SARS-CoV-2 Ag-RDTs was included. Additionally, our data on an overall impaired performance of Ag-RDTs at elevated temperatures are consistent across tests and analytical sensitivity for several tests was identical upon usage of either duplicates or higher numbers of replicates.

In sum, it was previously shown that virus concentrations of about 10^6^ genome copies per mL suffice for virus isolation and therefore serve as a correlate for infectivity [38,39]. Our study strongly suggests that short- and long-term exposure to elevated temperatures may compromise sensitivity of SARS-CoV-2 Ag-RDTs to an extent that may lead to false-negative test results at clinically relevant virus concentrations, potentially enhancing SARS-CoV-2 spread in tropical settings. At the same time, false-positive test results owed to test operation at low temperatures might not only lead to unwarranted individual quarantine assignments, but also to potential regional lockdown measures if those results were reported to public health authorities without confirmation by a gold standard test such as RT-PCR [20].

## Funding

This study is based on research funded in part by the Bill & Melinda Gates Foundation (grant ID INV-005971). The findings and conclusions contained within are those of the authors and do not necessarily reflect positions or policies of the Bill & Melinda Gates Foundation. The study was further supported in part by the Foundation for Innovative New Diagnostics (FIND), including procurement of some test kits.

## Author contributions

V.H.: conceptualization, investigation, validation, formal analysis, data curation, writing – original draft preparation, visualization. E.F.: methodology, investigation, validation. A.M.S.: investigation, validation, visualization. A.K.: investigation. C.F.: visualization, software. J.A.S.: methodology, resources, writing. V.M.C.: methodology. M.A.M.: methodology. C.D.: methodology. J.F.D.: conceptualization, methodology, resources, writing – original draft preparation, visualization, supervision, project administration, funding acquisition. All authors have read and agreed to the published version of the manuscript.

## Declaration of Competing Interest

The authors report no declarations of competing interest.
